# Diagnostic Accuracy of MUM1 (IRF4) Immunohistochemistry in Chronic Endometritis: A Systematic Review and Bayesian Meta-Analysis

**DOI:** 10.3390/diagnostics16081167

**Published:** 2026-04-15

**Authors:** Ana Maria Mihoci, Demetra Socolov, Eduard Cristian Mihoci, Loredana Maria Toma, Andreea Ioana Pruteanu, Răzvan Vladimir Socolov

**Affiliations:** 1 Grigore T. Popa University of Medicine and Pharmacy Iasi, 16 University Street, 700115 Iasi, Romania; demetra.socolov@umfiasi.ro (D.S.); mihoci.eduard-cristian@d.umfiasi.ro (E.C.M.); loredana-toma@umfiasi.ro (L.M.T.); andreea-ioana.pruteanu@umfiasi.ro (A.I.P.); 2 Clinical Hospital of Obstetrics and Gynecology “Elena Doamna”, 700038 Iasi, Romania; 3 Clinical Hospital of Obstetrics and Gynecology “Cuza Voda”, 700038 Iasi, Romania; 4 Department of Mother and Child Medicine, Grigore T. Popa University of Medicine and Pharmacy Iasi, 700115 Iasi, Romania; 5 Department of Medical Bioscience, Faculty of Bioengineering, Grigore T. Popa University of Medicine and Pharmacy Iasi, 700454 Iasi, Romania

**Keywords:** chronic endometritis, infertility, MUM1, IRF4, CD138, immunohistochemistry, diagnostic performance, Bayesian meta-analysis, plasma cells

## Abstract

**Highlights:**

**Abstract:**

**Background/Objectives:** Chronic endometritis (CE) is an underrecognized inflammatory disorder associated with infertility, recurrent pregnancy loss, and implantation failure. CD138 immunohistochemistry is widely used to detect endometrial plasma cells; however, background epithelial staining and interobserver variability may limit diagnostic precision. MUM1 (IRF4), a nuclear transcription factor expressed in plasma cells, has emerged as a potential complementary marker. We aimed to systematically evaluate the diagnostic performance of MUM1 immunohistochemistry relative to CD138-based histopathologic reference frameworks for chronic endometritis using a Bayesian meta-analytic framework. **Methods:** We conducted a systematic review and diagnostic test accuracy meta-analysis in accordance with PRISMA 2020 and PRISMA-DTA guidelines. MEDLINE, Embase, Scopus, and CENTRAL were searched from inception to 28 February 2026. Studies assessing MUM1 immunohistochemistry against predefined histopathologic reference frameworks for CE were eligible. Risk of bias was evaluated using QUADAS-2. A bivariate Bayesian random-effects model was applied to jointly estimate pooled sensitivity, specificity, likelihood ratios, and diagnostic odds ratio (DOR). **Results:** Six studies (*n* = 1574 women) were included in the qualitative synthesis, and four provided sufficient 2 × 2 data for quantitative pooling. The pooled sensitivity of MUM1 was 0.876 (95% credible interval (CrI): 0.536–0.976), and the pooled specificity was 0.853 (95% CrI: 0.653–0.930). The pooled positive likelihood ratio was 5.83 (95% CrI: 2.18–12.04), and the negative likelihood ratio was 0.15 (95% CrI: 0.03–0.56), corresponding to a DOR of 40.27 (95% CrI: 5.04–254.59). Credible intervals were wide, reflecting statistical uncertainty related to the limited number of studies and heterogeneity in diagnostic thresholds. **Conclusions:** MUM1 (IRF4) immunohistochemistry demonstrates potentially favorable comparative diagnostic performance relative to CD138-based reference frameworks, although substantial uncertainty remains due to limited and heterogeneous evidence. As an adjunctive nuclear marker, MUM1 may support histopathologic assessment of chronic endometritis; however, prospective head-to-head studies using harmonized diagnostic criteria and predefined plasma cell thresholds are required before routine implementation can be firmly recommended.

## 1. Introduction

Chronic endometritis (CE) represents a persistent inflammatory condition of the endometrial lining characterized by infiltration of plasma cells within the stromal compartment [1,2,3,4,5]. Although frequently asymptomatic, increasing evidence indicates that CE may adversely affect reproductive outcomes, including infertility, recurrent pregnancy loss (RPL), and recurrent implantation failure (RIF), highlighting its clinical relevance in reproductive medicine [6,7,8,9,10,11]. Reported prevalence varies substantially across studies, largely reflecting differences in diagnostic criteria, patient populations, and histopathological interpretation, underscoring the ongoing lack of standardization in diagnostic approaches [12,13,14,15].

Histopathologic identification of endometrial plasma cells remains the cornerstone of CE diagnosis [1,2,3,4,16]. CD138 immunohistochemistry is widely used to enhance plasma cell detection [2,3,17,18,19]; however, interobserver variability, inconsistent cut-off thresholds, and variability in staining protocols contribute to diagnostic heterogeneity [13,14,20,21,22]. In addition, CD138 expression may overlap with endometrial epithelial staining, potentially reducing diagnostic specificity and complicating interpretation [2,17].

MUM1 (multiple myeloma oncogene 1), also known as interferon regulatory factor 4 (IRF4), is a nuclear transcription factor expressed in late-stage B cells and plasma cells [20]. As a nuclear marker, MUM1 offers theoretical advantages in distinguishing true plasma cells from surrounding stromal elements and may improve diagnostic precision [20,21]. Emerging studies have evaluated MUM1 immunohistochemistry in CE, reporting improved plasma cell detection and promising concordance with CD138 compared with conventional approaches [21,22,23,24,25]. In some cohorts, MUM1-based diagnosis has been associated with reproductive outcomes, including frozen embryo transfer (FET) success rates and RIF prognosis [23,26,27].

In addition, advances in digital pathology and artificial intelligence-assisted plasma cell quantification may further enhance diagnostic reproducibility and reduce observer-related variability [21,23,28]. Despite these developments, findings remain heterogeneous, and diagnostic thresholds vary substantially across studies.

To date, no systematic review and Bayesian diagnostic meta-analysis has comprehensively assessed the comparative diagnostic performance and concordance of MUM1 in chronic endometritis. Therefore, the present study aimed to systematically evaluate the pooled sensitivity, specificity, and overall diagnostic performance of MUM1 using a bivariate Bayesian random-effects model [29,30].

## 2. Materials and Methods

### 2.1. Protocol and Registration

The present systematic review and Bayesian diagnostic meta-analysis were conducted in accordance with established international methodological standards for evidence synthesis, including the Preferred Reporting Items for Systematic Reviews and Meta-Analyses (PRISMA 2020) statement and its extension for diagnostic test accuracy studies (PRISMA-DTA) [31,32]. The PRISMA 2020 checklist is provided in the Supplementary Materials File S2.

The protocol was prospectively registered in the International Prospective Register of Systematic Reviews (PROSPERO; registration number CRD420261323577).

Risk of bias and methodological quality were assessed using the Quality Assessment of Diagnostic Accuracy Studies-2 (QUADAS-2) framework [33].

### 2.2. Eligibility Criteria

Eligibility criteria were predefined according to the PICO framework.

**Population**

Women of reproductive age undergoing evaluation for infertility, recurrent pregnancy loss, implantation failure, abnormal uterine bleeding, or suspected/confirmed chronic endometritis were eligible for inclusion.

**Index Test**

The index test was immunohistochemical detection of nuclear MUM1 (IRF4) expression in endometrial tissue samples.

**Reference Standard**

The reference standard was the histopathologic diagnosis of chronic endometritis, defined by the identification of endometrial plasma cells using CD138 immunohistochemistry and/or conventional histological assessment, according to each individual study.

**Outcomes**

The primary outcomes were pooled sensitivity and specificity. Secondary outcomes included positive likelihood ratio (LR+), negative likelihood ratio (LR−), and diagnostic odds ratio (DOR). Only studies providing sufficient data to reconstruct 2 × 2 contingency tables (true positives, false positives, true negatives, and false negatives) for MUM1 versus the reference standard were included in the quantitative meta-analysis.

**Study Design**

Eligible study designs included randomized controlled trials, prospective and retrospective cohort studies, case–control studies, and cross-sectional diagnostic accuracy studies.

**Exclusion Criteria**

Studies were excluded if they:

• Were case reports or case series including fewer than 10 patients;
• Were reviews, editorials, letters, or conference abstracts without full-text availability;
• Included animal or in vitro data;
• Did not provide extractable diagnostic accuracy data;
• Assessed MUM1 solely as a prognostic or therapeutic marker rather than as a diagnostic index test.

**Methodological Considerations Regarding the Reference Standard**

Chronic endometritis lacks a universally accepted gold standard. In most included studies, CD138 immunohistochemistry served as the primary reference standard; however, some investigations applied composite diagnostic frameworks incorporating histology, hysteroscopy, or molecular testing. Consequently, the present meta-analysis evaluates the relative diagnostic performance of MUM1 against heterogeneous reference frameworks rather than against a single independent disease-defining standard. This methodological characteristic reflects current clinical practice and the absence of a universally validated reference benchmark in this field.

### 2.3. Information Sources and Search Strategy

A systematic and comprehensive search of the literature was performed in the following electronic databases: MEDLINE (via PubMed), Embase, Scopus, and the Cochrane Central Register of Controlled Trials (CENTRAL). All records published from database inception until 28 February 2026 were considered eligible for screening.

The search strategy incorporated both controlled vocabulary terms (e.g., MeSH and Emtree) and relevant free-text keywords related to “chronic endometritis”, “MUM1”, “IRF4”, “immunohistochemistry”, “plasma cells”, and diagnostic test performance. In addition, reference lists of all included studies were manually examined to identify potentially relevant publications not captured through electronic database searching. No restrictions regarding language were applied.

Detailed search strategies for each database are provided in Supplementary Material File S1.

### 2.4. Screening and Eligibility Assessment

**Figure 1:**
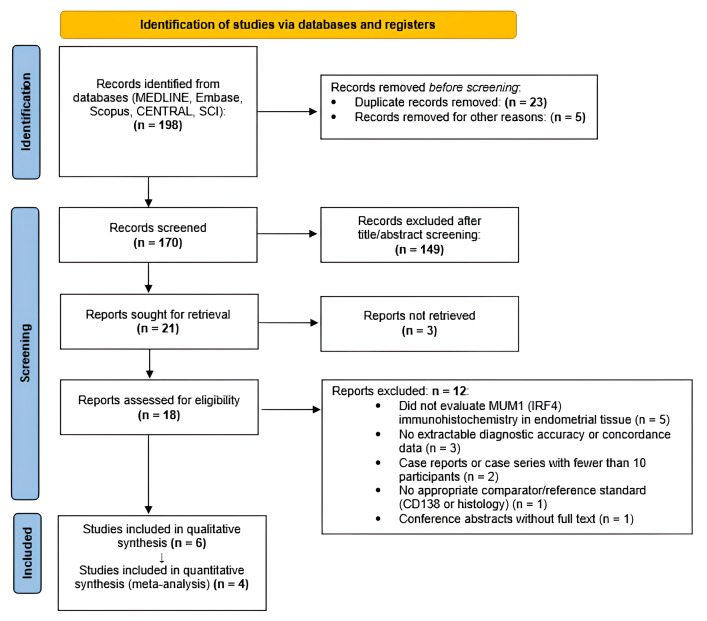
PRISMA 2020 flow diagram of study selection.

All retrieved records were imported into reference management software, where duplicate entries were identified and removed. Two reviewers independently screened titles and abstracts for potential eligibility. Full-text articles of potentially relevant studies were then assessed according to the predefined inclusion and exclusion criteria. Any disagreements between reviewers were resolved through discussion until consensus was reached. The overall study selection process is illustrated in Figure 1.

### 2.5. Data Extraction

Data extraction was performed independently by two reviewers using a standardized data collection form.

The following information was extracted:

• Study characteristics (first author, year, country, study design);
• Clinical context and patient population;
• Sample size;
• Details of the MUM1 immunohistochemistry protocol;
• Reference standard used;
• 2 × 2 contingency data (true positives, false positives, true negatives, and false negatives).

When necessary, diagnostic accuracy parameters were recalculated from the reported raw data.

### 2.6. Risk of Bias Assessment

Methodological quality and risk of bias were assessed using the Quality Assessment of Diagnostic Accuracy Studies-2 (QUADAS-2) tool. Risk of bias was evaluated across four domains:

1. Patient selection;
2. Index test;
3. Reference standard;
4. Flow and timing.

Each domain was judged as having low, high, or unclear risk of bias. Applicability concerns were also assessed.

### 2.7. Statistical Analysis

A bivariate Bayesian random-effects hierarchical model was applied to jointly estimate pooled sensitivity and specificity while accounting for the correlation between study-specific effects and between-study heterogeneity. Weakly informative prior distributions were specified to improve stability of parameter estimation in the context of sparse data. Normal prior distributions with a mean of 0 and a standard deviation of 1 were assigned to logit-transformed sensitivity and specificity parameters. Between-study standard deviations were assigned truncated normal prior distributions constrained to positive values. The correlation parameter between logit sensitivity and logit specificity was modeled using an LKJ(2) prior distribution. These prior specifications are consistent with recommended Bayesian hierarchical approaches for diagnostic test accuracy meta-analysis and allow robust estimation while appropriately reflecting uncertainty associated with limited evidence.

Posterior distributions were summarized using medians and 95% credible intervals (CrIs). Convergence of the Bayesian models was assessed using the diagnostics implemented in MetaBayesDTA, including visual inspection of trace plots and Gelman–Rubin R-hat statistics, with values below 1.1 indicating adequate convergence.

Derived summary measures included:

• Diagnostic odds ratio (DOR);
• Positive likelihood ratio (LR+);
• Negative likelihood ratio (LR−);
• False positive rate (1 − specificity).

Between-study heterogeneity was quantified using variance parameters for logit-transformed sensitivity and specificity, as well as the between-study correlation coefficient (ρ). A hierarchical summary receiver operating characteristic (HSROC) curve was constructed to illustrate overall diagnostic performance.

Descriptive forest plots were generated using Review Manager 5.4 (RevMan, The Cochrane Collaboration, Oxford, UK). Bayesian hierarchical modeling was performed using the MetaBayesDTA web application (version 1.5.3).

Given the limited number of included studies, formal subgroup or meta-regression analyses were not feasible. A predefined sensitivity analysis excluding the study using a composite genital tuberculosis-based reference standard was performed to explore the potential impact of heterogeneous reference frameworks on pooled estimates.

Given the heterogeneity of reference standards across studies, pooled estimates should be interpreted as measures of comparative diagnostic performance relative to existing diagnostic frameworks rather than as indicators of absolute disease verification accuracy.

### 2.8. Ethical Considerations

As this study was based exclusively on previously published data, ethical approval and informed consent were not required.

## 3. Results

### 3.1. Study Selection

A total of 198 records were identified through database searching. After the removal of 23 duplicate records and 5 records excluded for other reasons, 170 records underwent title and abstract screening. Of these, 149 records were excluded. Twenty-one reports were sought for full-text review; three reports could not be retrieved. Eighteen full-text articles were assessed for eligibility. Twelve studies were excluded for the following reasons: five did not evaluate MUM1 (IRF4) immunohistochemistry in endometrial tissue, four did not provide extractable diagnostic accuracy data, and three were case reports or case series including fewer than 10 patients. Six studies met the inclusion criteria and were included in the qualitative synthesis. Of these, four provided sufficient data for inclusion in the quantitative meta-analysis.

The study selection process is illustrated in Figure 1.

### 3.2. Study Characteristics

The main characteristics of the included studies are presented in Table 1. The six studies were published between 2019 and 2023 and comprised a total of 1574 participants. Study designs included prospective and retrospective cohort studies, as well as multicenter analyses.

Clinical contexts included infertility, recurrent implantation failure, recurrent pregnancy loss, abnormal uterine bleeding, and suspected or confirmed chronic endometritis. All studies evaluated MUM1 (IRF4) immunohistochemistry in endometrial tissue samples. In most studies, CD138 immunohistochemistry served as the reference standard, whereas one study used a composite reference standard including laparoscopy, histology, and molecular testing.

Diagnostic thresholds for plasma cell positivity varied across studies, ranging from ≥1 plasma cell per high-power field to ≥5 plasma cells per defined tissue section, potentially contributing to methodological heterogeneity.

### 3.3. Risk of Bias

The methodological quality and risk of bias assessment, performed using the QUADAS-2 tool, is summarized in Table 2. Two studies (Klimaszyk (2023) [7]; Cicinelli (2022) [22]) were judged to have a low risk of bias across all domains. In contrast, three studies demonstrated a high risk of bias in the reference standard domain, primarily due to potential incorporation bias or lack of independent confirmation.

Concerns regarding patient selection were identified in two studies, mainly related to retrospective design or selective inclusion criteria. Applicability concerns were rated as high in two studies and moderate in two others, largely reflecting variations in clinical context and reference standards.

Overall, the methodological quality of the included studies was considered moderate, with heterogeneity primarily attributable to differences in reference standards and diagnostic thresholds.

### 3.4. Diagnostic Accuracy of Individual Studies

**Figure 2:**
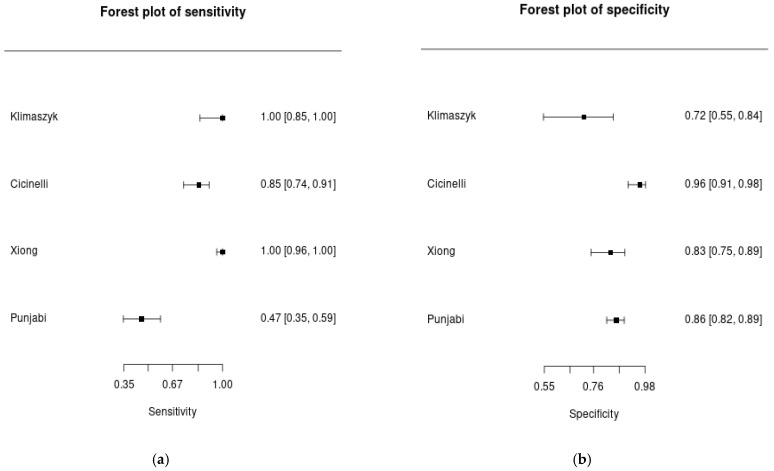
Forest plots of pooled diagnostic performance estimates for MUM1 (IRF4) immunohistochemistry in chronic endometritis. (**a**) Sensitivity and (**b**) specificity estimates. Squares represent individual study estimates, with horizontal lines indicating 95% credible intervals (CrI). The pooled estimates were derived using a bivariate Bayesian random-effects model [6,7,22,23].

Individual diagnostic performance estimates are presented in Table 3 and Figure 2. Sensitivity ranged from 46.7% to 100% across studies. Two studies (Klimaszyk (2023) [7]; Xiong (2023) [23]) reported a sensitivity of 100%, whereas Punjabi (2023) [6] reported a lower sensitivity of 46.7% in the context of genital tuberculosis-associated chronic endometritis.

Specificity ranged from 71.9% to 95.9%. The highest specificity was observed in the multicenter study by Cicinelli (2022) [22], while lower specificity was reported by Klimaszyk (2023) [7].

Overall, variability was greater for sensitivity than for specificity, suggesting heterogeneity in case definitions and diagnostic thresholds across studies. Four studies provided sufficient 2 × 2 contingency data for inclusion in the quantitative meta-analysis.

### 3.5. Pooled Diagnostic Performance

Using a bivariate Bayesian random-effects model, the pooled sensitivity of MUM1 (IRF4) immunohistochemistry for the diagnosis of chronic endometritis was 0.876 (95% credible interval (CrI): 0.536–0.976), while the pooled specificity was 0.853 (95% CrI: 0.653–0.930). The pooled positive likelihood ratio (LR+) was 5.83 (95% CrI: 2.18–12.04), and the pooled negative likelihood ratio (LR−) was 0.15 (95% CrI: 0.03–0.56). The diagnostic odds ratio (DOR) was 40.27 (95% CrI: 5.04–254.59), which is suggestive of favorable overall comparative diagnostic performance, although the wide credible interval reflects uncertainty related to the limited number of included studies.

**Figure 3:**
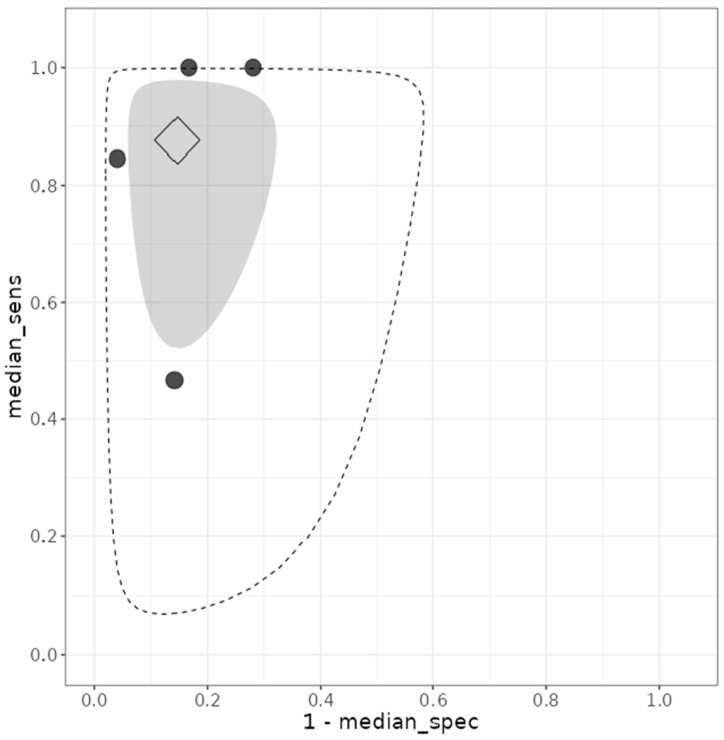
Hierarchical summary receiver operating characteristic (HSROC) curve of MUM1 (IRF4) immunohistochemistry for the diagnosis of chronic endometritis. Each circle represents an individual study estimate. The diamond indicates the pooled estimate derived from the bivariate Bayesian random-effects model. The shaded inner region represents the 95% credible region, and the outer dashed contour corresponds to the 95% prediction region.

Between-study heterogeneity was greater for sensitivity (between-study standard deviation for logit-transformed sensitivity, σ_1_ = 1.903) than for specificity (σ_0_ = 0.846). The between-study correlation coefficient (ρ = −0.154) suggested a weak negative correlation between sensitivity and specificity across studies. The hierarchical summary receiver operating characteristic (HSROC) curve is presented in Figure 3 and illustrates the overall diagnostic performance of MUM1 across included studies.

A sensitivity analysis excluding the study using a composite genital tuberculosis-based reference standard yielded a pooled sensitivity of 0.941 (95% CrI: 0.659–0.987) and a pooled specificity of 0.854 (95% CrI: 0.577–0.950). These estimates overlapped with the primary analysis, indicating that inclusion of the GTB-associated cohort did not materially influence overall diagnostic performance. The higher pooled sensitivity in this restricted analysis primarily reflects the lower sensitivity observed in the GTB-associated cohort rather than the instability of the Bayesian model. The pooled diagnostic performance estimates are summarized in Table 4.

**Table 4:** Pooled diagnostic performance of MUM1 (IRF4).

Diagnostic Parameter	Pooled Estimate	95% Credible Interval (CrI)
Sensitivity	0.876	0.54–0.98
Specificity	0.853	0.65–0.93
Positive likelihood ratio (LR+)	5.83	2.18–12.04
Negative likelihood ratio (LR−)	0.15	0.03–0.56
Diagnostic odds ratio (DOR)	40.27	5.04–254.59

## 4. Discussion

### 4.1. Principal Findings

In this systematic review and Bayesian diagnostic meta-analysis, MUM1 (IRF4) immunohistochemistry showed promising pooled sensitivity (0.876; 95% CrI: 0.536–0.976) and specificity (0.853; 95% CrI: 0.653–0.930) for the diagnosis of chronic endometritis. The pooled diagnostic odds ratio (40.27; 95% CrI: 5.04–254.59) suggests meaningful discriminatory capacity, although the wide credible interval reflects substantial statistical imprecision related to the small number of available studies.

The pooled positive likelihood ratio (5.83) indicates that a positive MUM1 result is associated with an increased probability of classification within CD138-based diagnostic frameworks for chronic endometritis. The negative likelihood ratio (0.15) suggests a meaningful reduction in disease probability when the test is negative. Although between-study heterogeneity was observed, particularly for sensitivity, overall diagnostic performance remained consistently favorable across studies. Collectively, these findings provide preliminary quantitative support for further evaluation of MUM1 within standardized histopathological diagnostic frameworks.

### 4.2. Context Within Existing Literature

Chronic endometritis has also been associated with impaired implantation and adverse reproductive outcomes in several observational and interventional studies, including reports evaluating antibiotic treatment strategies and their impact on subsequent pregnancy rates [12,13,34,35,36,37]. In addition, hysteroscopic findings such as endometrial micropolyps and refined histological diagnostic criteria have been proposed to improve disease characterization and standardize clinical diagnosis [5,12,38].

The broader immunological and inflammatory microenvironment of the endometrium has further been implicated in the pathophysiology of chronic endometritis, supporting its role in reproductive dysfunction and implantation failure [26,30,31,36,39,40,41]. Recent investigations have also emphasized the contribution of plasma cells as key immunological mediators in IVF failure, underscoring the need for precise and reproducible diagnostic markers in reproductive medicine [27,42].

Early immunohistochemical observations exploring plasma cell markers in endometrial tissue laid the groundwork for subsequent investigations into alternative nuclear markers such as MUM1 [43].

### 4.3. Clinical Implications

Accurate detection of chronic endometritis is highly relevant in reproductive medicine, given its reported associations with infertility, recurrent pregnancy loss, and implantation failure [6,7,8,9,10,11,44]. Improved diagnostic precision may therefore influence patient stratification, therapeutic decision-making, and counseling in selected clinical contexts.

The positive likelihood ratio observed in this analysis suggests that a positive MUM1 result may be associated with a meaningful increase in post-test probability of chronic endometritis, whereas the low negative likelihood ratio indicates potential value in reducing disease probability when findings are negative. However, these estimates should be interpreted in light of the variability in plasma cell thresholds and reference standards across studies, which limits immediate generalizability and underscores the need for harmonized diagnostic criteria prior to widespread clinical implementation [13,24].

Importantly, because CD138 immunohistochemistry functioned as the reference standard in most included studies, the present meta-analysis assesses the diagnostic performance of MUM1 relative to an established comparator rather than against an independent disease-defining benchmark. Accordingly, the findings should not be interpreted as evidence of superiority but rather as supporting a potentially non-inferior or complementary role within current diagnostic frameworks.

In clinical practice, MUM1 may therefore be considered an adjunctive nuclear marker, particularly in cases with equivocal CD138 staining, background epithelial reactivity, or interpretative uncertainty [21,22]. Nonetheless, definitive conclusions regarding replacement, superiority, or incremental diagnostic benefit require prospectively designed, blinded head-to-head studies to apply uniform and predefined diagnostic thresholds.

At present, the available evidence does not establish standardized diagnostic thresholds for MUM1 interpretation and does not provide sufficient prospective validation to support routine use as a standalone diagnostic marker. MUM1 should therefore currently be regarded as a complementary marker that may assist histopathological interpretation in selected clinical contexts, particularly in situations where background epithelial CD138 staining or interpretative variability complicate plasma cell identification.

### 4.4. Strengths and Limitations

This study represents, to our knowledge, the first systematic review and Bayesian diagnostic meta-analysis specifically evaluating MUM1 (IRF4) immunohistochemistry in chronic endometritis. A key methodological strength is the application of a bivariate Bayesian random-effects model [29,30], which jointly estimates sensitivity and specificity while accounting for between-study heterogeneity and their correlation. This approach is particularly appropriate when only a small number of studies are available, as it allows direct quantification of uncertainty through posterior distributions and reduces the risk of overprecision in sparse datasets. The limited number of studies available for quantitative synthesis represents an important constraint on the interpretation of pooled estimates. Accordingly, the wide credible intervals observed for sensitivity, specificity, and derived summary measures reflect substantial statistical uncertainty related to sparse data and between-study heterogeneity. These findings should therefore be interpreted cautiously and regarded as exploratory and hypothesis-generating rather than definitive estimates of comparative diagnostic performance.

The review was conducted in accordance with established methodological standards for systematic reviews and diagnostic accuracy studies [31,32,33], including prospective protocol registration and structured QUADAS-2 assessment.

Several limitations should be acknowledged. Although six studies were included in the qualitative synthesis, only four provided extractable 2 × 2 contingency data for quantitative pooling. This limited evidence base constrained statistical precision and precluded formal subgroup or meta-regression analyses. The relatively wide credible intervals observed for pooled sensitivity and specificity therefore reflect inherent uncertainty rather than deficiencies of the modeling framework.

Between-study heterogeneity, particularly for sensitivity, likely reflects differences in clinical populations, staining protocols, interpretative criteria, and plasma cell cut-off thresholds. Such variability may indicate a threshold effect contributing to differences in diagnostic performance across studies.

Variability in plasma cell cut-off thresholds used to define chronic endometritis may influence observed sensitivity and specificity by altering the balance between diagnostic inclusiveness and specificity. Lower thresholds may increase sensitivity by identifying milder inflammatory infiltrates but may also increase the risk of misclassification of physiological inflammatory changes. Conversely, higher thresholds may improve specificity but may fail to detect low-grade inflammatory processes. Differences in threshold definitions may therefore contribute to between-study heterogeneity and partially explain variability in pooled sensitivity estimates observed across included studies.

An important methodological consideration concerns the heterogeneity of reference standards. Chronic endometritis lacks a universally accepted gold standard. While CD138 immunohistochemistry served as the primary comparator in most studies, some investigations employed composite diagnostic frameworks incorporating histology, hysteroscopy, or molecular testing. Consequently, pooled estimates represent relative diagnostic performance compared with heterogeneous diagnostic frameworks rather than validation against an independent disease-defining benchmark. This may introduce incorporation or verification bias and should be considered when interpreting accuracy estimates.

The absence of universally harmonized histopathological criteria represents a fundamental limitation in chronic endometritis research. Although CD138 immunohistochemistry is widely used for plasma cell identification, variability in diagnostic thresholds and interpretative criteria contributes to heterogeneity across studies. Therefore, pooled estimates should be interpreted as measures of concordance with currently applied diagnostic frameworks rather than absolute indicators of disease verification accuracy [12,13,14,45].

The inclusion of heterogeneous clinical contexts—including infertility-associated chronic endometritis, recurrent implantation failure, recurrent pregnancy loss, and infection-related endometrial inflammation—may also contribute to variability in observed performance. However, given the limited number of available studies, restriction to narrower subgroups would have substantially reduced interpretability.

Importantly, this meta-analysis does not establish whether MUM1 provides incremental diagnostic value beyond CD138 in standardized, blinded head-to-head comparisons using uniform thresholds, nor does it define an optimal plasma cell cut-off for routine implementation. These questions require prospectively designed studies, ideally structured as non-inferiority or equivalence investigations using harmonized methodological criteria.

### 4.5. Future Directions

Future prospective multicenter studies using standardized plasma cell thresholds and clearly defined reference standards are warranted [12,13,14]. In particular, predefined cut-offs (e.g., ≥5 plasma cells per high-power field) should be applied consistently to reduce interpretative variability and improve comparability across studies. Blinded, same-slide head-to-head comparisons between MUM1 and CD138 within identical patient populations would provide more definitive evidence regarding their relative diagnostic performance [20,24,25].

Prospective investigations specifically designed as non-inferiority or equivalence studies are needed to determine whether MUM1 offers incremental value beyond CD138 or may serve as a reliable alternative in routine practice. Integration of digital pathology and artificial intelligence-assisted plasma cell quantification may further enhance reproducibility and reduce observer-dependent variability in histopathological assessment [23,28].

In addition, future research should aim to evaluate MUM1 within more homogeneous clinical subgroups, such as isolated recurrent implantation failure or infertility without infectious etiologies, to clarify context-specific diagnostic performance. Harmonization of histological criteria, hysteroscopic findings, and molecular diagnostic approaches may ultimately facilitate the development of standardized diagnostic algorithms for chronic endometritis in reproductive medicine [5,27,38,46].

## 5. Conclusions

MUM1 (IRF4) immunohistochemistry demonstrates promising relative diagnostic performance compared with CD138-based diagnostic frameworks in this Bayesian meta-analysis. However, the limited number of available studies and the absence of a universally accepted reference standard introduce substantial uncertainty, reflected by wide credible intervals. Current evidence primarily supports the role of MUM1 as an adjunctive marker that may support histopathological interpretation in selected clinical scenarios. Further prospective, standardized head-to-head studies using harmonized diagnostic thresholds are required before routine clinical implementation can be definitively recommended.

## Figures and Tables

**Table 1:** General features of included studies.

Study ID	Country	Study Design	Population (*N*)	Clinical Context	Index Test	Reference Standard	CE Diagnostic Threshold	Key Findings
Klimaszyk (2023) [7]	Poland	Observational	54 women with RPL	Recurrent pregnancy loss	MUM1 IHC	CD138 IHC	≥1 plasma cell/10 HPF	MUM1 identified a higher proportion of CE cases than CD138 (57.4% vs. 40.7%); moderate agreement (κ = 0.60).
Parks (2019) [21]	USA	Retrospective	311 endometrial biopsies	Suspected CE/AUB	MUM1 IHC	CD138 IHC; H&E histology	≥1 plasma cell	Higher plasma-cell detection with MUM1 (48%) compared with CD138 (23%) and H&E (15%); reduced epithelial background staining.
Xiong (2023) [23]	China	Observational	298 infertile women	Infertility evaluation	Dual CD138/MUM1 IHC with AI-assisted counting	Conventional CD138 IHC with manual plasma-cell counting	>5 plasma cells per section	Dual staining improved diagnostic accuracy and reduced misclassification compared with CD138 alone.
Punjabi (2023) [6]	India	Retrospective	391 infertile women	Genital tuberculosis-associated CE	MUM1 IHC	Composite GTB diagnosis (laparoscopy, histology, GeneXpert)	≥1 plasma cell	Sensitivity 46.7% and specificity 85.8% for GTB-associated CE; high negative predictive value.
Cicinelli (2022) [22]	Italy	Multicenter retrospective	193 women	Infertility/RPL/AUB	MUM1 IHC	CD138 IHC (primary comparison); hysteroscopy + histology (ROC analysis)	≥1 plasma cell/20 HPF	Higher diagnostic accuracy and interobserver reproducibility compared with CD138.
Li (2023) [8]	China	Retrospective	327 women with RIF	Recurrent implantation failure	MUM1/CD138 IHC	Post-treatment reassessment and clinical outcomes	≥5 plasma cells/30 HPF	CE was associated with reduced FET outcomes; resolution after treatment improved reproductive outcomes.

**Table 2:** Risk of bias and applicability concerns.

Study	Patient Selection	Index Test	Reference Standard	Flow and Timing	Applicability Concerns
Klimaszyk (2023) [7]	Low	Low	Low	Low	Low
Cicinelli (2022) [22]	Low	Low	Low	Low	Low
Parks (2019) [21]	High	Low	High	Low	Moderate
Xiong (2023) [23]	Low	Unclear	High	Low	High
Punjabi (2023) [6]	High	Low	High	Low	High
Li (2023) [8]	Low	Low	High	Low	Moderate

**Table 3:** Diagnostic accuracy (2 × 2 contingency data) of MUM1 (IRF4) immunohistochemistry for the diagnosis of chronic endometritis.

Study	Population (*N*)	Reference Standard	MUM1 Cut-Off	TP	FP	FN	TN	Sensitivity (%)	Specificity (%)
Klimaszyk (2023) [7]	54	CD138 IHC	≥1 plasma cell/10 HPF	22	9	0	23	100	71.9
Cicinelli (2022) [22]	193	CD138 IHC	≥1 plasma cell/20 HPF	60	5	11	117	84.5	95.9
Xiong (2023) [23] *	198	Manual CD138 diagnosis	>5 plasma cells per section	96	17	0	85	100	83.3
Punjabi (2023) [6] **	391	Composite GTB diagnosis	≥1 plasma cell	28	47	32	284	46.7	85.8

* For Xiong (2023) [23], only cases with manual CD138 diagnosis used as a reference standard were included in the 2 × 2 analysis. ** Punjabi (2023) [6] used a composite reference standard for genital tuberculosis-associated chronic endometritis (laparoscopy, histology, and molecular testing).

## Data Availability

The data analyzed in this study are derived from previously published studies and are available within the article and its Supplementary Materials.

## Supplementary Materials

The following supporting information can be downloaded at https://www.mdpi.com/article/10.3390/diagnostics16081167/s1. File S1: Full electronic search strategies for all databases; File S2: PRISMA 2020 checklist.

## Abbreviations

The following abbreviations are used in this manuscript:

AI	Artificial Intelligence
AUB	Abnormal Uterine Bleeding
CD138	Cluster of Differentiation 138
CE	Chronic Endometritis
CrI	Credible Interval
DOR	Diagnostic Odds Ratio
FET	Frozen Embryo Transfer
FN	False Negative
FP	False Positive
GTB	Genital Tuberculosis
H&E	Hematoxylin and Eosin
HPF	High-Power Field
HSROC	Hierarchical Summary Receiver Operating Characteristic
IHC	Immunohistochemistry
IRF4	Interferon Regulatory Factor 4
LR+	Positive Likelihood Ratio
LR−	Negative Likelihood Ratio
MUM1	Multiple Myeloma Oncogene 1
PRISMA	Preferred Reporting Items for Systematic Reviews and Meta-Analyses
PRISMA-DTA	Preferred Reporting Items for Systematic Reviews and Meta-Analyses for Diagnostic Test Accuracy
PROSPERO	International Prospective Register of Systematic Reviews
QUADAS-2	Quality Assessment of Diagnostic Accuracy Studies-2
RIF	Recurrent Implantation Failure
RPL	Recurrent Pregnancy Loss
ROC	Receiver Operating Characteristic
TN	True Negative
TP	True Positive
