# Supplementary material for: Diagnostic Accuracy of MUM1 (IRF4) Immunohistochemistry in Chronic Endometritis: A Systematic Review and Bayesian Meta-Analysis
Source: Diagnostics (Basel). 2026 Apr 15;16(8):1167. doi: 10.3390/diagnostics16081167 (PMC13115080; doi:10.3390/diagnostics16081167)
Supplement: Supplementary file 1 [file diagnostics-16-01167-s001.zip › S1_Search_Strategies.pdf]

Supplementary Material S1

Detailed Search Strategies

1. MEDLINE (via PubMed)

Search date: 28 February 2026
Database coverage: From inception to 28 February 2026

The following search strategy was used:

("chronic endometritis"[MeSH Terms] OR "chronic endometritis"[Title/Abstract]
OR "endometrial inflammation"[Title/Abstract])
AND
("MUM1"[Title/Abstract] OR "IRF4"[Title/Abstract]
OR "interferon regulatory factor 4"[Title/Abstract])
AND
("immunohistochemistry"[MeSH Terms]
OR "immunohistochemistry"[Title/Abstract]
OR "plasma cells"[Title/Abstract])
AND
("diagnostic accuracy"[Title/Abstract]
OR sensitivity[Title/Abstract]
OR specificity[Title/Abstract]
OR "diagnostic performance"[Title/Abstract])

No language restrictions were applied.

2. Embase

Search date: 28 February 2026
Database coverage: From inception to 28 February 2026

The following search strategy was used:

('chronic endometritis'/exp
OR 'chronic endometritis':ti,ab
OR 'endometrial inflammation':ti,ab)
AND
('mum1':ti,ab

OR 'irf4':ti,ab
OR 'interferon regulatory factor 4':ti,ab)
AND
('immunohistochemistry'/exp
OR 'immunohistochemistry':ti,ab
OR 'plasma cell'/exp)
AND
('diagnostic accuracy':ti,ab
OR sensitivity:ti,ab
OR specificity:ti,ab
OR 'diagnostic performance':ti,ab)

No language restrictions were applied.

3. Scopus

Search date: 28 February 2026
Database coverage: From inception to 28 February 2026

The following search strategy was used:

TITLE-ABS-KEY ("chronic endometritis" OR "endometrial inflammation")
AND
TITLE-ABS-KEY ("MUM1" OR "IRF4" OR "interferon regulatory factor 4")
AND
TITLE-ABS-KEY ("immunohistochemistry" OR "plasma cells")
AND
TITLE-ABS-KEY ("diagnostic accuracy" OR sensitivity OR specificity
OR "diagnostic performance")

No language restrictions were applied.

4. Cochrane CENTRAL

Search date: 28 February 2026
Database coverage: From inception to 28 February 2026

The following search strategy was used:

("chronic endometritis" OR "endometrial inflammation")
AND
("MUM1" OR "IRF4" OR "interferon regulatory factor 4")
AND
("immunohistochemistry" OR "plasma cells")
AND
("diagnostic accuracy" OR sensitivity OR specificity
OR "diagnostic performance")

No language restrictions were applied.
