# Supplementary material for: Diagnostic Accuracy of MUM1 (IRF4) Immunohistochemistry in Chronic Endometritis: A Systematic Review and Bayesian Meta-Analysis
Source: Diagnostics (Basel). 2026 Apr 15;16(8):1167. doi: 10.3390/diagnostics16081167 (PMC13115080; doi:10.3390/diagnostics16081167)
Supplement: Supplementary file 1 [file diagnostics-16-01167-s001.zip › S2_PRISMA_2020_Checklist.pdf]

PRISMA 2020 Checklist

Section and Topic	Item #	Checklist item	Location where item is reported
**TITLE**			
Title	1	Identify the report as a systematic review.	Title page
**ABSTRACT**			
Abstract	2	See the PRISMA 2020 for Abstracts checklist.	Abstract
**INTRODUCTION**			
Rationale	3	Describe the rationale for the review in the context of existing knowledge.	Introduction (Section 1)
Objectives	4	Provide an explicit statement of the objective(s) or question(s) the review addresses.	Introduction (final paragraph)
**METHODS**			
Eligibility criteria	5	Specify the inclusion and exclusion criteria for the review and how studies were grouped for the syntheses.	Methods Section 2.2 (Eligibility Criteria)
Information sources	6	Specify all databases, registers, websites, organisations, reference lists and other sources searched or consulted to identify studies. Specify the date when each source was last searched or consulted.	Methods Section 2.3 (Information Sources and Search Strategy)
Search strategy	7	Present the full search strategies for all databases, registers and websites, including any filters and limits used.	Supplementary Material S1 (Full electronic search strategies)
Selection process	8	Specify the methods used to decide whether a study met the inclusion criteria of the review, including how many reviewers screened each record and each report retrieved, whether they worked independently, and if applicable, details of automation tools used in the process.	Methods Section 2.4 (Study Selection)
Data collection process	9	Specify the methods used to collect data from reports, including how many reviewers collected data from each report, whether they worked independently, any processes for obtaining or confirming data from study investigators, and if applicable, details of automation tools used in the process.	Methods Section 2.5 (Data Extraction)
Data items	10a	List and define all outcomes for which data were sought. Specify whether all results that were compatible with each outcome domain in each study were sought (e.g. for all measures, time points, analyses), and if not, the methods used to decide which results to collect.	Methods Section 2.2 (Eligibility Criteria – Outcomes) and Results Section 3.5
	10b	List and define all other variables for which data were sought (e.g. participant and intervention characteristics, funding sources). Describe any assumptions made about any missing or unclear information.	Methods Section 2.5 (Data Extraction)
Study risk of bias assessment	11	Specify the methods used to assess risk of bias in the included studies, including details of the tool(s) used, how many reviewers assessed each study and whether they worked independently, and if applicable, details of automation tools used in the process.	Methods Section 2.6 (Risk of Bias Assessment –

Section and Topic	Item #	Checklist item	Location where item is reported
			QUADAS-2)
Effect measures	12	Specify for each outcome the effect measure(s) (e.g. risk ratio, mean difference) used in the synthesis or presentation of results.	Methods Section 2.7 (Statistical Analysis)
Synthesis methods	13a	Describe the processes used to decide which studies were eligible for each synthesis (e.g. tabulating the study intervention characteristics and comparing against the planned groups for each synthesis (item #5)).	Methods Section 2.7 (Statistical Analysis)
	13b	Describe any methods required to prepare the data for presentation or synthesis, such as handling of missing summary statistics, or data conversions.	Methods Section 2.7 (Statistical Analysis)
	13c	Describe any methods used to tabulate or visually display results of individual studies and syntheses.	Methods Section 2.7 and Figures 2–3
	13d	Describe any methods used to synthesize results and provide a rationale for the choice(s). If meta-analysis was performed, describe the model(s), method(s) to identify the presence and extent of statistical heterogeneity, and software package(s) used.	Methods Section 2.7 (Statistical Analysis)
	13e	Describe any methods used to explore possible causes of heterogeneity among study results (e.g. subgroup analysis, meta-regression).	Methods Section 2.7 (Statistical Analysis)
	13f	Describe any sensitivity analyses conducted to assess robustness of the synthesized results.	Methods Section 2.7 (Sensitivity Analysis)
Reporting bias assessment	14	Describe any methods used to assess risk of bias due to missing results in a synthesis (arising from reporting biases).	Not formally assessed due to the limited number of included studies (<10), which precluded reliable evaluation of reporting bias.
Certainty assessment	15	Describe any methods used to assess certainty (or confidence) in the body of evidence for an outcome.	Not formally assessed
**RESULTS**			
Study selection	16a	Describe the results of the search and selection process, from the number of records identified in the search to the number of studies included in the review, ideally using a flow diagram.	Results Section 3.1 and Figure 1 (PRISMA Flow Diagram)
	16b	Cite studies that might appear to meet the inclusion criteria, but which were excluded, and explain why they were excluded.	Results Section 3.1
Study characteristics	17	Cite each included study and present its characteristics.	Results Section 3.2 and Table 1
Risk of bias in studies	18	Present assessments of risk of bias for each included study.	Results Section 3.3 and Table 2
Results of individual studies	19	For all outcomes, present, for each study: (a) summary statistics for each group (where appropriate) and (b) an effect estimate and its precision (e.g. confidence/credible interval), ideally using structured tables or plots.	Results Section 3.4 and Table 3

PRISMA 2020 Checklist

Section and Topic	Item #	Checklist item	Location where item is reported
Results of syntheses	20a	For each synthesis, briefly summarise the characteristics and risk of bias among contributing studies.	Results Sections 3.2 and 3.3
	20b	Present results of all statistical syntheses conducted. If meta-analysis was done, present for each the summary estimate and its precision (e.g. confidence/credible interval) and measures of statistical heterogeneity. If comparing groups, describe the direction of the effect.	Results Section 3.5 and Table 4
	20c	Present results of all investigations of possible causes of heterogeneity among study results.	Results Section 3.5
	20d	Present results of all sensitivity analyses conducted to assess the robustness of the synthesized results.	Results Section 3.5 (Sensitivity analysis)
Reporting biases	21	Present assessments of risk of bias due to missing results (arising from reporting biases) for each synthesis assessed.	Not assessed due to limited number of studies
Certainty of evidence	22	Present assessments of certainty (or confidence) in the body of evidence for each outcome assessed.	Certainty of evidence was not formally assessed (e.g., using GRADE) due to the limited number and heterogeneity of available studies.
**DISCUSSION**			
Discussion	23a	Provide a general interpretation of the results in the context of other evidence.	Discussion Section 4.1 (Principal Findings)
	23b	Discuss any limitations of the evidence included in the review.	Discussion Section 4.4 (Strengths and Limitations)
	23c	Discuss any limitations of the review processes used.	Discussion Section 4.4 (Strengths and Limitations)
	23d	Discuss implications of the results for practice, policy, and future research.	Discussion Sections 4.3 and 4.5
**OTHER INFORMATION**			
Registration and protocol	24a	Provide registration information for the review, including register name and registration number, or state that the review was not registered.	Methods Section 2.1 (PROSPERO registration number: CRD420261323577)
	24b	Indicate where the review protocol can be accessed, or state that a protocol was not prepared.	Methods Section 2.1
	24c	Describe and explain any amendments to information provided at registration or in the protocol.	No amendments reported
Support	25	Describe sources of financial or non-financial support for the review, and the role of the funders or sponsors in the review.	Funding section
Competing	26	Declare any competing interests of review authors.	Conflicts of Interest

PRISMA 2020 Checklist

Section and Topic	Item #	Checklist item	Location where item is reported
interests			section
Availability of data, code and other materials	27	Report which of the following are publicly available and where they can be found: template data collection forms; data extracted from included studies; data used for all analyses; analytic code; any other materials used in the review.	Data Availability Statement

From: Page MJ, McKenzie JE, Bossuyt PM, Boutron I, Hoffmann TC, Mulrow CD, et al. The PRISMA 2020 statement: an updated guideline for reporting systematic reviews. BMJ 2021;372:n71. doi: 10.1136/bmj.n71. This work is licensed under CC BY 4.0. To view a copy of this license, visit https://creativecommons.org/licenses/by/4.0/
